# Satisfaction with life and psychological distress during the COVID-19 pandemic: An Egyptian online cross-sectional study

**DOI:** 10.4102/phcfm.v14i1.2896

**Published:** 2022-01-31

**Authors:** Ahmed H. El-Monshed, Ahmed Loutfy, Moustafa T. Saad, Ahmed S. Ali, Abdel-Hady El-Gilany, Ahmed Soliman Mohamed, Mahmoud Salah, Mohamed Zoromba

**Affiliations:** 1 Department of Psychiatric and Mental Health Nursing, Faculty of Nursing, Mansoura University, Mansoura, Egypt; 2 Department of Paediatric Nursing, Faculty of Nursing, Beni-Suef University, Beni-Suef, Egypt; 3 Mohammed Al-Mana College for Medical Sciences (MACHS), Dammam, KSA; 4 Department of Gerontological Nursing, Faculty of Nursing, Mansoura University, Mansoura, Egypt; 5 Department of Paediatric Nursing, Faculty of Nursing, Mansoura University, Mansoura, Egypt; 6 Department of Public Health and Preventive Medicine, Faculty of Medicine, Mansoura University, Mansoura, Egypt; 7 Hamad Medical Corporation (HMC), Doha, Qatar; 8 Surgical Specialty Center (SSC), Doha, Qatar

**Keywords:** COVID-19, Egypt, life satisfaction, pandemic, psychological distress

## Abstract

**Background:**

Coronavirus disease 2019 (COVID-19) is a novel sickness that emerged worldwide as an unprecedented crisis and led to major effects on the daily life of the general public as well as negative impacts on their mental well-being.

**Aim:**

This study aimed to assess satisfaction with life and psychological distress during the COVID-19 pandemic in Egypt.

**Setting:**

An online study was conducted in Egypt.

**Methods:**

A cross-sectional online survey was fulfilled by 1056 Egyptian adults from 06 to 13 June 2020. Psychological distress and satisfaction with life were measured by Arabic validated versions of the Kessler Psychological Distress Scale (K10) and the Satisfaction with Life Scale (SWLS).

**Results:**

About half of the surveyed respondents (51%) were satisfied with their life, whilst 57.4% experienced severe psychological distress. The independent predictors of satisfaction with life are being married, satisfactory income, low distress, moderate distress and high distress (adjusted odds ratio [AOR] = 1.2, 3.0, 2.5, 6.9, 5.2 and 2.1, respectively). Being a female, having secondary education, > secondary education, unsatisfactory income and presence of mental illness are the independent predictors of mental distress (AOR = 2.3, 3.9, 1.9, 1.9, 1.6 and 4.0, respectively).

**Conclusion:**

The study provides evidence about the high prevalence of psychological distress during the peak period of Egypt’s COVID-19 pandemic. The study results highlight the enhancement of development interventions to promote psychological well-being and feeling of satisfaction with life during the pandemic.

## Introduction

Since December 2019, the novel coronavirus disease 2019 (COVID-19) has spread from Wuhan city to different areas in China and around the world.^1^ On 11 March 2020, the World Health Organization (WHO) declared the COVID-19 outbreak a pandemic.^2^ On 14 February 2020, the Egyptian Ministry of Health reported the first case of COVID-19. To control the COVID-19 outbreak, the Egyptian government introduced preventive and containment measures with a partial closure starting on 25 March. On 31 March, Egypt pronounced 710 COVID-19 cases and 46 related deaths with a mortality rate of 6.48%.^3,4^ As on 06 June 2020, there were 1497 new cases with total cases of 32 612, a total of 1198 deaths and 8538 full recovery.^5^

Generally, several potential stressors were caused by the pandemic that might lead to psychological distress and life dissatisfaction.^6^ Individuals’ overall assessments of their psychological well-being and quality of life are referred to as life satisfaction.^7^ Psychological distress takes the shape of a negative emotion, which contrasts with life contentment. It refers to people’s unfavourable emotional reactions to a number of stimuli, which might include tension, dread, worry and psychological instability.^8^

Fear of contracting COVID-19 and the implications for oneself or loved ones might be potential stressors linked to the virus. The measure taken to prevent the spread of the virus have a number of drawbacks, including social isolation, economic costs, disruption of people’s work and lifestyles and anxiety about the future. As a result, it is reasonable to expect a rise in psychological discomfort and negative implications for the mental health of vast populations throughout the world.^9,10^

Several early studies provided evidence regarding psychological distress in the context of the COVID-19 pandemic. According to an online survey of the general public in China, more than over half of those polled assessed the psychological impact of the events as moderate-to-severe, with 16.5% reporting depressed symptoms and 28.8% reporting anxiety symptoms.^1^ In a follow-up survey 4 weeks later, these proportions appeared to be rather steady, with no substantial reduction in those symptoms.^11^

Another study looking at the emotional indicators before and after the declaration of COVID-19 found that negative emotions increased, whilst the scores of positive emotions and life satisfaction decreased.^12^

Studies investigating the psychological effects of prior epidemics or pandemics such as the Ebola epidemic in 2014^13,14^ or the severe acute respiratory syndrome (SARS) outbreak in 2003,^15^ found that they were linked to significant psychological discomfort and mental health problems. Given that the COVID-19 pandemic brought additional obstacles for patients, it was suggested that psychiatric nursing interventions be re-adapted to address COVID-19-related concerns as well. Fear, worry and uncertainty about one’s own and the health of one’s family escalated as a result of the pandemic. Furthermore, COVID-19 exposed people to their own and others’ suffering and some of them experienced terrible events and there were the unfortunate ones who lost their lives as a result of the pandemic. Furthermore, patients and caregivers were not permitted to interact in person.^16^

Educative interventions supplied patients (and carers during the calls) with up-to-date and realistic information about the coronavirus disease as a first step in the COVID-related mental nursing intervention. During the pandemic, patients with anxiety and acute stress symptoms were given individual relaxation methods such as muscle relaxation, body scan, breath control and creative relaxation.^17^ Irrational beliefs and maladaptive interpretations were challenged and modified through cognitive restructuring.^18^

Given the novelty of the COVID-19 pandemic, the precautionary measures implemented to contain the spread of the disease, and the lack of published research regarding the coronavirus in Egypt, it was important to highlight whether the COVID-19 pandemic had affected life satisfaction of the Egyptian people and has caused psychological distress as an initial step for proposing nursing interventions for the people.

## Research design and method

### Study design

A cross-sectional online survey was conducted within the predictable peak (one week from 06 to 13 June 2020) month of the COVID-19 outbreak in Egypt.

### Setting

This study was conducted in Egypt, a densely populated country (approximately more than 100 millions inhabitants in 2020). Internet is now available with considerable connectivity and availability and is used extensively by educated people of all ages. Many studies were performed online because of the COVID-19 lockdown and people are familiar with this method of data collection.

### Population and sampling strategy

The target population included adults (18 years and above) in all regions of Egypt who were willing to participate in the study. The study adopted an online survey and the study population may not reflect the actual pattern of the general population. Sample size was calculated using Medcalc 15.8 (https://www.medcalc.org/). The primary outcome of interest is the percentage of people with satisfaction with life. An internal pilot study on 100 subjects found that 51.0% of them were satisfied with their life. With Alpha error of 5.0%, the study power of 90.0% and 5.0% precision, the sample size is 1047 subjects.

### Tools and data collection

Data were collected anonymously through an online semi-structured Arabic questionnaire created using Google Forms™, with a valid link for one week and comprised three parts including a consent form for all respondents.

#### Socio-demographic and personal characteristics

Characteristics included gender, age, marital status, having children, governorate, residence, level of education, employment status, number of family members, household income, presence of any chronic diseases, presence of any psychiatric disorders in any family member or one of the respondents’ friends, relatives or neighbours who were infected with COVID-19 and what are the sources of the respondents’ knowledge regarding COVID-19.

#### The Kessler Psychological Distress Scale (K10)

A 10-item 5-point scale (10–50 score) is a self-report questionnaire used for measuring the levels of psychological distress.^19^ The respondents choose the most relevant response for them in the last 4 weeks. After summing scores, the range of 10–15, 16–21, 22–29 and 30–50 represent low, moderate, high and very high psychological distress, respectively.^20^ It was reported that the reliability of K10 was high with Cronbach’s alpha of 0.93.^21^ Besides another study stated that the internal consistency of Arabic K10 was also high with Cronbach’s alpha of 0.88.^22^

#### Satisfaction with Life Scale

A self-report questionnaire intended to determine the level of satisfaction with life composed of 5-item rated on 7-point scale.^23^ After summing scores, the range of 5–9, 10–14, 15–19, 21–25, 26–30 and above 31 reflect extremely dissatisfied, dissatisfied, slightly dissatisfied, slightly satisfied, satisfied and extremely satisfied with life, respectively. A score of 20 reflects the impartial point on the scale. The Satisfaction with Life Scale (SWLS) has good internal consistency (Cronbach’s alpha = 0.80).^24^ The Arabic version of SWLS was used in the present study with overall good internal consistency (Cronbach’s alpha = 0.79 and test-retest reliability of 0.83).^25^

### Procedure

In Egypt, the government restrictions implemented to decrease the spread of COVID-19 through banning gatherings held up the study all over the country, therefore a web-based survey was required. The obscure online survey link was sent through emails and shared on two of the most commonly used social networking sites (Facebook and WhatsApp) within one week (06 to 13 June 2020). The authors asked respondents to send the survey to their friends and family (snowball technique). The average completion time of the survey was 5–8 min.

### Data analysis

Data were analysed using the Statistical Package for the Social Science (SPSS) version 23. Categorical variables were presented as numbers and percentages. Chi-square was used to test for significant differences between groups. Crude odds ratio (COR) and their 95% confidence intervals (95% CI) were calculated. Factors significantly associated with satisfaction with life and severe psychological distresses were entered into a multivariate logistic regression model using the Wald stepwise forward method. Adjusted odds ratio (AOR) and their 95% CI were calculated. A value of *p* ≤ 0.05 was considered statistically significant.

### Ethical considerations

Ethical approval was obtained from the Research Ethics Committee of Faculty of Nursing, Mansoura University (reference number: P.0200). Thereafter, the population fulfilling the eligibility criteria and having filled the informed consent, could open the link and participate in the study. No monetary rewards were given for completing the questionnaire.

### Results

Table 1 shows that participants with older age, married, having children, those employed, family size less than five members and satisfactory family income are associated with higher likelihood of satisfaction with life (COR = 1.9, 4.5, 1.7, 1.9, 1.4 and 2.5, respectively). Being a female, divorced/widowed, completed at least a secondary education and with unsatisfactory family income, increases the likelihood of a severe degree of psychological distress (COR = 2.4, 4.7, 1.8, 1.8 and 1.7, respectively).

**TABLE 1:** Prevalence of satisfaction with life and severe psychological distress amongst study participants and their variation with the socio-demographic factors.

Socio-demographic factors	Total	Satisfaction with life				Severe distress			
		*n*	%	COR	95% CI	*n*	%	COR	95% CI
Overall	1056	359	51.0	-	-	606	57.4	-	-
**Age (years)**									
< 20	88	36	40.9	1	*r*	55	62.5	1.4	0.8–2.2
20–29	687	343	49.9	1.4	0.9–2.3	396	57.6	1.1	0.8–1.5
30 >	281	160	56.9**	1.9	1.2–3.1	155	55.2	1	*r*
**Gender**									
Male	282	144	51.1	1	*r*	118	41.8	1	*r*
Female	774	395	51.0	0.99	0.8–1.3	488	63.0***	2.4	1.8–3.1
**Marital status**									
Single	647	291	45.0*	2.0	1.1–4.1	364	56.3	1	*r*
Married	367	236	64.3***	4.5	2.2–9.1	206	56.1	0.99	0.8–1.3
Widow/divorced	42	12	28.6	1	*r*	36	85.7***	4.7	1.9–11.2
**Having children**									
No	697	325	46.6	1	*r*	392	56.2	1	*r*
Yes	359	214	59.6***	1.7	1.3–2.2	214	59.6	1.1	0.9–1.5
**Residence**									
Urban	564	302	53.5	1	*r*	324	57.4	1	*r*
Rural	492	237	48.2	0.8	0.6–1.0	282	57.3	1.0	0.8–1.3
**Geographic region**									
Lower Egypt	652	328	50.3	1	*r*	384	58.9	1	*r*
Frontiers	115	65	56.5	1.3	0.8–1.9	75	65.2	1.3	0.9–2.0
Upper Egypt	289	146	50.5	1.0	0.8–1.3	147	50.9	0.7	0.5–1
**Education**									
< 2 years	123	73	59.3*	1.6	1.1–2.4	55	44.7	1	*r*
2 years	616	313	50.8	1.1	0.8–1.5	365	59.3**	1.8	1.2–2.7
> 2 years	317	153	48.3	1	*r*	186	58.7**	1.8	1.2–2.9
**Occupation**									
Private work†	277	147	53.1*	1.3	1.1–1.8	148	53.4	1	*r*
Employee	178	108	60.7**	1.9	1.3–2.6	101	56.7	1.1	0.8–1.7
Housewife/other	174	90	51.7	1.3	0.9–1.8	99	56.9	1.2	0.8–1.7
Students	427	194	45.4	1	*r*	250	60.4	1.2	0.9–1.7
**Family size**									
< 5	425	236	55.5**	1.4	1.1–1.7	246	57.9	1	*r*
5 >	631	303	48.0	1	*r*	360	57.1	1.0	0.8–1.2
**Family income**									
Satisfactory	789	448	56.8***	2.5	1.9–3.4	429	54.4	1	*r*
Unsatisfactory	267	91	34.1	1	*r*	177	66.3***	1.7	1.2–2.2

COR, crude odds ratio; CI, confidence interval; *r*, reference category.

*, **, *** , Significant difference compared with the reference category at ≤ 0.05, ≤ 0.01 and ≤ 0.001, respectively.

† , Work in the private sector and not affiliated with the government sector.

As presented in Table 2, the absence of mental illness, having no friends infected with COVID-19, low psychological distress, moderate psychological distress and high psychological distress are associated with increased satisfaction with life (COR = 2.1, 1.3, 7.0, 5.1 and 2, respectively). Having a mental illness is associated with a more severe degree of distress (COR = 3.8).

**TABLE 2:** Prevalence of satisfaction with life and severe psychological distress amongst study participants and their variation with the clinical and mental factors.

Clinical and mental factors	Total	Satisfaction with life				Severe distress			
		*n*	%	COR	95% CI	*n*	%	COR	95% CI
**Chronic diseases**									
No	955	486	50.9	1	*r*	542	56.8	1	*r*
Yes	101	53	52.5	1.1	0.7–1.6	64	63.4	1.3	0.9–2.0
**Mental illness**									
No	992	517	52.1***	2.1	1.2–3.5	553	55.7	1	*R*
Yes	64	22	34.4	1	*r*	53	82.8***	3.8	2.0–7.4
**Family member infected**									
No	1034	526	50.4	1	*r*	591	57.2	1	*r*
Yes	22	13	59.1	1.4	0.6–3.3	15	68.2	1.6	0.6–4.0
**Infected friend**									
No	732	389	53.1*	1.3	1.1–1.7	709	55.9	1	*R*
Yes	324	150	46.3	1	*R*	197	60.8	1.2	0.9–1.6
**Psychological distress**									
Low	197	146	74.1***	7.0	4.7–10.7	-	-	-	-
Moderate	253	171	67.6***	5.1	3.6–7.3	-	-	-	-
High	282	128	45.4***	2.0	1.4–2.8	-	-	-	-
Very high	324	94	29.0	1	*r*	-	-	-	-

COR, crude odds ratio; CI, confidence interval; *r*, reference category.

*, **, *** , Significant difference compared with the reference category at ≤ 0.05, ≤ 0.01 and ≤ 0.001, respectively.

The multivariate logistic regression analysis presented in Table 3 revealed that the independent predictors of life satisfaction are being married, satisfactory income, low distress, moderate distress and high psychological distress (AOR = 1.2, 3.0, 2.5, 6.9, 5.2 and 2.1, respectively). Being a female, having secondary education, > secondary education, unsatisfactory income and presence of mental illness are the independent predictors of psychological distress with AOR of 2.3, 3.9, 1.9, 1.9, 1.6 and 4.0, respectively.

**TABLE 3:** Multivariable logistic regression analysis of independent predictors of satisfaction with life and severe distress.

Independent predictors	Satisfaction with life				Severe distress			
	β	*p*	AOR	95% CI	β	*p*	AOR	95% CI
**Gender**								
Male	-	-	-	-	-	≤ 0.001	1	*r*
Female	-	-	-	-	0.8	-	2.3	1.7–3.1
**Marital status**								
Single	0.1	0.7	1.2	0.5–2.5	0.02	0.8	1	*r*
Married	1.1	0.005	3.0	1.4–6.4	1.4	0.003	1.0	0.8–1.3
Widow/divorced	-	-	1	*r*	-	-	3.9	1.6–9.7
**Education**								
< Secondary	-	-	-	-	-	-	1	*r*
Secondary	-	-	-	-	0.7	0.002	1.9	1.3–2.9
> Secondary	-	-	-	-	0.7	0.004	1.9	1.2–3.0
**Family income**								
Satisfactory	0.9	≤ 0.001	2.5	1.8–3.5	0.5	0.001	1	*r*
Unsatisfactory	-	-	1	*r*	-	-	1.6	1.2–2.2
**Mental illness**								
No	-	-	-	-	-	≤ 0.001	1	*r*
Yes	-	-	-	-	1.4	-	4.0	2.0–7.8
**Psychological distress**								
Low	1.9	≤ 0.001	6.9	4.6–10.5	-	-	-	-
Moderate	1.7	≤ 0.001	5.2	3.6–7.6	-	-	-	-
High	0.9	≤ 0.001	2.1	1.5–2.9	-	-	-	-
Very high	-	-	1	*r*	-	-	-	-

Note: Constant: satisfaction with life = –2.1, severe distress = –1.1; Model χ^2^: satisfaction with life = 216.8 and *p* ≤ 0.001; Model χ^2^: severe distress = 91.3, *p* ≤ 0.001; % correctly predicted: satisfaction with life = 71.0, satisfaction with life = 63.6.

AOR, adjusted odds ratio; CI, confidence interval; *r*, reference category.

## Discussion

Any massive pandemic will have a negative impact on the community. Individuals respond to major infectious disease pandemic emotionally and display high levels of uncertainty and distress.^26^ The results of this study indicate that around half (51.0%) of the surveyed respondents were satisfied with their life whilst 57.4% of them experienced severe psychological distress. Plausible explanations could be that this study was conducted within the peak month of Egypt’s COVID-19 outbreak. In an epidemic, people display common stress reactions such as fear of getting ill and passing away, fear of being ill and dying, fear of being unable to work during isolation, fear of being fired from their job and losing their money, dread of being quarantined, feeling powerless to protect their family and fear of loved ones dying as a result of the virus.^11^ Because possibilities to communicate face-to-face socially are limited during lockdown, subjective sentiments of loneliness have increased dramatically.^27^

Indeed, the pandemic is associated with an especially high toll when it comes to how individuals feel about their social relationships and their health with reports of loneliness and depression doubling, tripling or even quintupling over the prior known rates.^28^ Egyptian government imposed preventive measures including closing the borders, establishing a state of emergency accompanied by a curfew from 20:00 to 06:00, ban on all gatherings, closing of schools and universities, which had a strong impact on daily workers, commerce, crafts and the informal sector. Under these circumstances, the COVID-19 pandemic has a strong negative impact on happiness and life satisfaction and in turn, people often experienced high levels of psychological distress.

Prior research had demonstrated immediate negative emotional impact in response to the COVID-19 pandemic.^1,12,29,30,31,32^ An Egyptian study suggested that 23.9% of the respondents experienced a mild level of psychological impact, whilst 52.0% demonstrated moderate and severe levels of psychological impact.^29^ In addition, lower rates were demonstrated in an Italian study but in the early stage of the pandemic which found that only 38% of the Italian population displayed degrees of psychological distress after short exposure to the pandemic.^30^ Besides, a nationwide study conducted during the COVID-19 pandemic on approximately 52 000 participants from 36 Chinese provinces revealed that about 35.0% of the participants had psychological distress.^31^

In April 2020, an international online research conducted in seven languages found that before the lockdown more than 60% of respondents agreed to be content with their lives, whereas just 30% agreed during the lockdown. The total score on life satisfaction assessments dropped by 16%, with more individuals feeling unsatisfied ‘during’ the lockdown period than ‘before’.^33^ In a similar line, Chinese research showed that COVID-19’s social distancing tactics resulted in lower life satisfaction and increased sadness.^10^

On studying the associations between the respondents’ socio-demographic factors and the studied measures, the study results showed that being a female increased the likelihood of a severe degree of distress. According to a previous study females, on average, are more prone to loneliness, anxiety and depression than males.^34^ This might be because females are more sensitive emotionally. These results concurs with prior research studies, which showed that females are, to a certain degree, more vulnerable to experience psychological distress during the COVID-19 pandemic.^1,29,30,35^ By contrast, a cross-sectional survey in China that studied the effect of the COVID-19 outbreak on local residents’ psychological well-being found that there was not any association between gender and psychological well-being.^36^

People whose level of education was more than secondary school were most likely to experience higher levels of psychological distress, probably because of their high self-awareness of the COVID-19 pandemic and of their health. A similar result was reported in a Chinese study, which found that highly educated people had higher rates of distress.^31^

As financial concerns have an important role in several decisions of daily life, they are likely to cause recurrent or consistent daily hassles. In line with this, we found that unsatisfactory income in this study population was significantly associated with high scores of psychological distress and low scores of life satisfaction.

These results indicated that individuals who are more satisfied with their life changes during the COVID-19 pandemic were less distressed and vice versa. Maybe these individuals had the ability to rationalise or justify their inactive lifestyles and adapt effectively with social distancing measures and then became less frustrated by the restrictive measures during the pandemic. There is no doubt that during a life crisis, people need to rearrange their priorities and to create major behavioural readjustments in their daily lifestyle.^37^ Overloads of such changes because of the COVID-19 pandemic during short periods of time may have severe burdens on individuals’ adjustment abilities and then affect their psychological well-being. In addition, financial problems caused by the COVID-19 pandemic severely limit people’s life options and develop a feeling of fear and uncertainty for them, their families, and their current and future prospects.

In general, growing literature has documented that high degrees of satisfaction with life are associated with high physical and psychological well-being and various aspects of high social and cognitive functioning.^38,39,40^ This results from this study are in agreement with those reported by Zhang et al. who confirmed that people who were more satisfied with their life changes suffered less distress during the COVID-19 pandemic.^10^ Moreover, in Turkey, another study demonstrated that the fear of COVID-19 negatively correlated with life satisfaction.^41^ In addition, results of a study on 317 respondents who participated during the beginning of the dynamic increase of the outbreak in Poland mentioned that anxiety and COVID-19 stress were negatively associated with life satisfaction.^42^

## Limitations of the study

Despite the study’s relevance and merits, such as the timing of data collection during Egypt’s COVID-19 peak, it had significant flaws. Firstly, the study used an online survey because of the restricted resources available and the COVID-19 pandemic’s temporal sensitivity and the study population did not reflect the general population’s true pattern. Secondly, the study’s fundamental nature, such as the sample approach being limited to persons with internet access, may limit generalisability because of the difficulties of contacting people who do not utilise network devices or who are unable to read and write. Finally, there was selection bias because of oversampling of a certain network of responders (e.g. respondents aged between 20 and 29 years, females, respondents from lower Egypt).

## Conclusion

In conclusion, this study demonstrates the significant incidence of psychological distress during Egypt’s COVID-19 pandemic’s expected peak phase. During the pandemic, this research raises some serious issues regarding life satisfaction. Conducting a prospective research on vulnerable populations such as children, the elderly and the uneducated will be beneficial. In addition, to avoid additional psychosocial issues and reduce psychological distress, a complete crisis prevention and intervention system should be developed, which includes epidemiological surveillance, screening, referral and focused intervention. Furthermore, national strategic planning and coordination for psychological first aid during large-scale pandemics should be established, with a focus on women. The findings of the study also imply that programmes aimed at enhancing psychological well-being in the general population should be developed quickly, taking into account people’s individual features and histories. Finally, longitudinal studies should be used in the future to uncover protective and risk variables for psychological distress during post-epidemic periods.
